# Transcriptome and metabolome analyses of two contrasting sesame genotypes reveal the crucial biological pathways involved in rapid adaptive response to salt stress

**DOI:** 10.1186/s12870-019-1665-6

**Published:** 2019-02-11

**Authors:** Yujuan Zhang, Donghua Li, Rong Zhou, Xiao Wang, Komivi Dossa, Linhai Wang, Yanxin Zhang, Jingyin Yu, Huihui Gong, Xiurong Zhang, Jun You

**Affiliations:** 1 Key Laboratory of Biology and Genetic Improvement of Oil Crops of the Ministry of Agriculture and Rural Affairs, Oil Crops Research Institute of the Chinese Academy of Agricultural Sciences, Wuhan, 430062 China; 2 Cotton Research Center, Shandong Academy of Agricultural Sciences, Jinan, 250100 China; 3 Centre d’Etude Régional pour l’Amélioration de l’Adaptation à la Sécheresse (CERAAS), Route de Khombole, 3320 Thiès, BP Senegal

**Keywords:** Salt stress, Sesame, Transcriptome, Metabolome, Metabolic pathway, Amino acid, Raffinose

## Abstract

**Background:**

Soil salinity is one of the major serious factors that affect agricultural productivity of almost all crops worldwide, including the important oilseed crop sesame. In order to improve salinity resistance in sesame, it is crucial to understand the molecular mechanisms underlying the adaptive response to salinity stress.

**Results:**

In the present study, two contrasting sesame genotypes differing in salt tolerance were used to decipher the adaptive responses to salt stress based on morphological, transcriptome and metabolome characterizations. Morphological results indicated that under salt stress, the salt-tolerant (ST) genotype has enhanced capacity to withstand salinity stress, higher seed germination rate and plant survival rate, as well as better growth rate than the salt-sensitive genotype. Transcriptome analysis revealed strongly induced salt-responsive genes in sesame mainly related to amino acid metabolism, carbohydrate metabolism, biosynthesis of secondary metabolites, plant hormone signal transduction, and oxidation-reduction process. Especially, several pathways were preferably enriched with differentially expressed genes in ST genotype, including alanine, aspartate and glutamate metabolism, carotenoid biosynthesis, galactose metabolism, glycolysis/gluconeogenesis, glyoxylate and dicarboxylate metabolism, porphyrin and chlorophyll metabolism. Metabolome profiling under salt stress showed a higher accumulation degree of metabolites involved in stress tolerance in ST, and further highlighted that the amino acid metabolism, and sucrose and raffinose family oligosaccharides metabolism were enhanced in ST.

**Conclusions:**

These findings suggest that the candidate genes and metabolites involved in crucial biological pathways may regulate salt tolerance of sesame, and increase our understanding of the molecular mechanisms underlying the adaptation of sesame to salt stress.

**Electronic supplementary material:**

The online version of this article (10.1186/s12870-019-1665-6) contains supplementary material, which is available to authorized users.

## Background

Soil salinity as one of the major serious abiotic stresses, occur mainly in coastal and arid/semi-arid regions and affects plant life processes limiting the agricultural productivity and distribution of crops around the world [1, 2]. Currently, more than 20% of the cultivated land (1000 million ha) is salt affected, and this number is increasing due to global climatic changes and poor management of irrigation and applied fertilizers [2]. Developing salt-tolerant and high yielding plant varieties is the most efficient way to prevent the yield loss in plant production. It is imperative to understand the salinity response and tolerance mechanisms of plants, which helps us develop traditional breeding and biotechnological approaches to improve stress resistance in plants.

High salinity causes hyperosmotic stress, oxidative stress, ionic imbalance, Na^+^ toxicity and even death in plants [3, 4]. To tolerate the salt stress condition, plants have developed a series of morphological, physiological, biochemical and molecular adjustment mechanisms to keep growth, development and productivity [5]. These acclimation responses include regulation of osmotic adjustment, ion homeostasis, signaling transduction, and induction of antioxidative enzymes activities, etc. [6, 7]. Salt tolerance is a complex trait governed by genetic factors. Many salt-responsive genes, which functions involved in regulation of ion accumulation and exclusion, stress signal transduction, transcription regulation, redox reactions, and accumulation of specific osmoregulation substances, have been identified to play important roles in salt tolerance in many plant species [8]. Transgenic plants achieved by overexpression of some salt tolerance genes exhibits enhanced resistance to salt in different degrees. These genes include *AlSAP* (an A20/AN1 zinc-finger gene) [9], *BADH* (betaine aldehyde dehydrogenase gene) [10], *CCD1* (a gene encoding calcium-binding protein with a C-terminal centrin-like domain) [11], *PtVP1.1* (H^+^-pyrophosphatase gene) [12], *mtlD* (a gene for mannitol biosynthesis) [13], *NHX* (a Na^+^/H^+^ antiporter gene) [14], *P5CS* (delta^1^-pyrroline-5-carboxylate synthetase gene) [15], *SOS1* (*Salt Overly Sensitive* pathway gene) [16], *OsbZIP23* (basic leucine zipper transcription factor gene) [17]. It’s worth noting that durum wheat grain yield on saline soils is increasing by 25% by introgression of an ancestral Na^+^ transporter gene *TmHKT1;5-A* in the *Nax2* locus from *Triticum monococcum* [18]. These studies clearly demonstrated that improvements in plant salt tolerance by gene transfer and marker-assisted breeding is highly possible and desirable.

Sesame (*Sesamum indicum* L.) is one of the oldest oilseed crops worldwide, and considered as an excellent health food for human [19]. Knowledge of the importance of sesame in human nutrition is increasing following reports of the antioxidative activity, antiaging effect, antihypertensive activity, reducing inflammation and atherosclerosis, and other health-related functions of sesame oil and seed components [19, 20]. In recent years, the demand for high quality sesame seeds in the international market was increased, but the current yield and quality production of sesame cannot meet the increasing demand of the international market [21]. Compared with most of the oilseed crops, sesame is rated relatively salt and drought tolerant crop which should be spread into more coastal and arid/semi-arid areas to increase global sesame production [22]. At present, sesame salt tolerance varieties are urgently needed in these salt-affected zones to overcome salinity effects, especially in Xinjiang and Inner Mongolia regions of China and several African countries. Several studies have mainly focused on the physiological and biochemical responses to salt stress and the identification of some salt-tolerance candidate genes [22–24]. However, there is a lack of scientific studies on the salt tolerance molecular mechanisms and the relationship between the transcriptomic responses and the metabolomic responses to salt stress which are needed to facilitate the developing salt-tolerant sesame varieties. For this study, the transcriptome and metabolome profiles in the seedlings of salt-tolerant and sensitive sesame genotypes were performed in the early phase of salt stress. This study revealed crucial metabolic pathways and metabolites in response to salt stress between the two contrasting genotypes, providing important insights into the mechanisms underlying sesame salt adaptation and tolerance.

## Results

### Phenotypic differences between WZM3063 and ZZM4028 in response to salinity stress

**Fig. 1:**
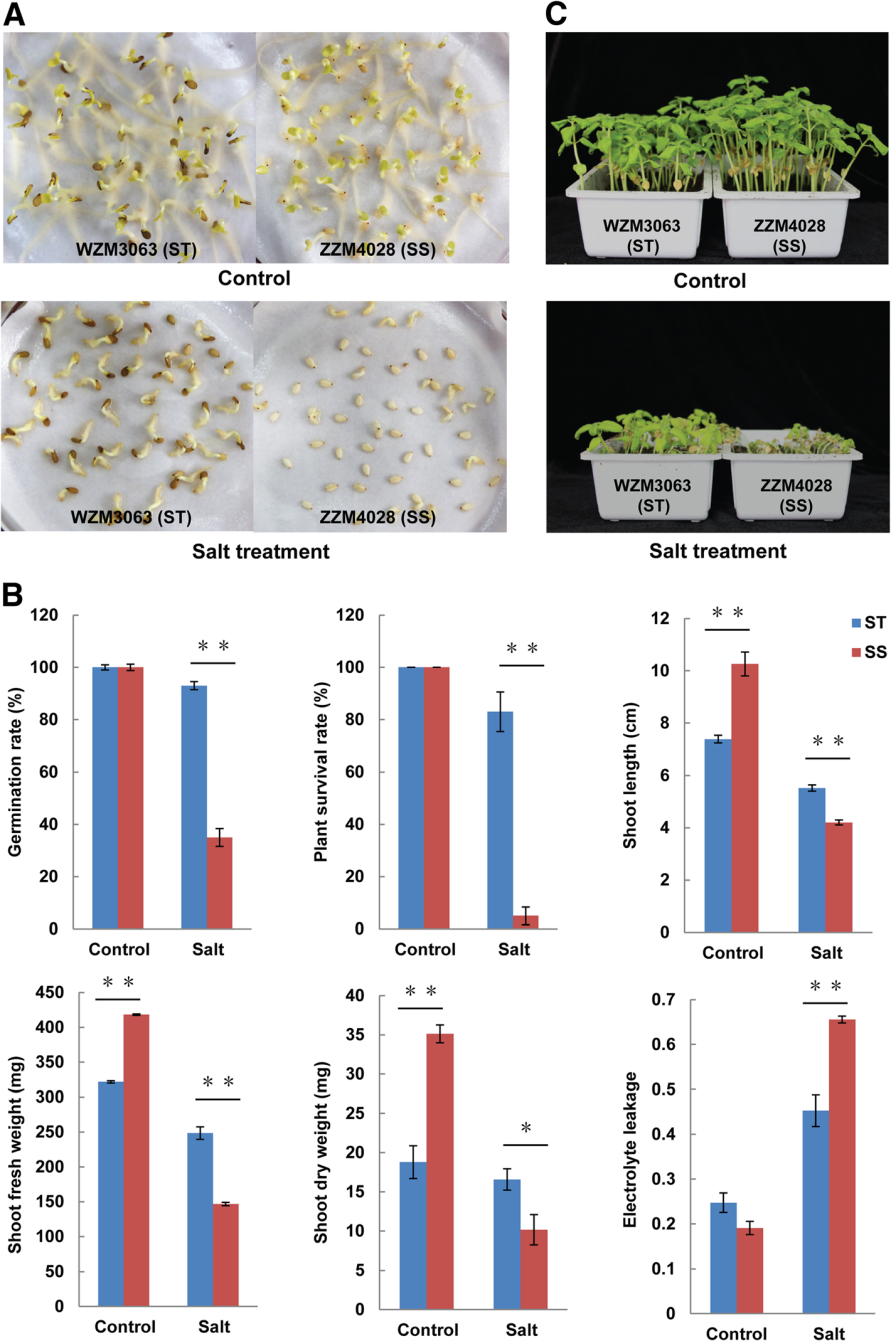
Morphological changes of salt tolerant (ST) and salt sensitive (SS) in response to salinity stress. **a** Germination of ST and SS under control and 150 mM NaCl treatments. **b** Seed germination rate, plant survival rate, shoot length, shoot fresh weight, shoot dry weight and relative electrolyte leakage of ST and SS under control and salt conditions. **c** Growth of ST and SS was monitored when 6-days-old seedlings were subjected to 100 mM NaCl for 21d. Bars with asterisk (*) are significantly different (∗*P* < 0.05, ∗∗*P* < 0.01, two-tailed Student’s *t* tests) between the two genotypes

Seed germination rates of WZM3063 (salt-tolerant, ST) and ZZM4028 (salt-sensitive, SS) genotypes under control and salt conditions were primarily measured and were found to be more severely affected for SS genotype than ST genotype at 150 mM NaCl concentration (Fig. 1a and b). Similarly, the capacity to withstand salinity stress was significantly weaker in SS genotype seedlings than ST. ST and SS seedlings exposure to salt stress (100 mM NaCl) led to leaf chlorosis, leaf tip burning, severe wilting, stunted growth and even to death, but the injury degree was lesser for ST than SS (Fig. 1c). Although differences were observed between ST and SS for shoot length and shoot weight under the control condition, the values of plant survival rate, shoot length and shoot dry weight of SS genotype were reduced much more strongly in SS than ST under salt condition (Fig. 1b). Leaf electrolyte leakage, as indicator of membrane damage, was significantly higher in the SS than in ST under salt stress, suggesting more membrane damage during salt stress in the SS genotype. All these results confirmed that ST genotype withstands salinity stress much better than SS genotype.

### Transcriptomic analysis of ST and SS in response to salt stress

#### Overview of the transcriptomic responses of ST and SS to salt stress

**Fig. 2:**
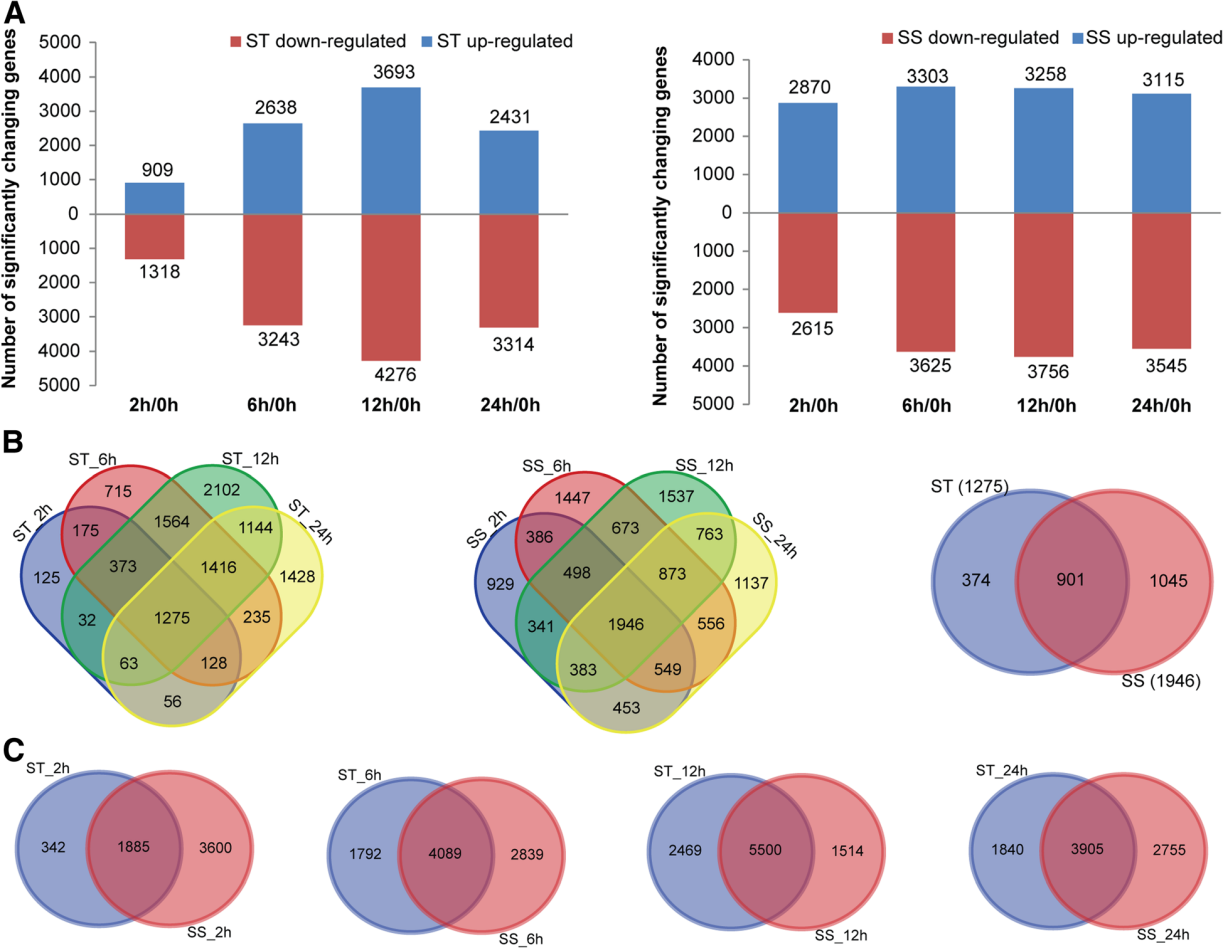
Differentially expressed genes (DEGs) in ST and SS in response to salt stress. **a** Numbers of DEGs in ST and SS at different salt stress time points. Venn diagrams of DEGs among different salt stress time points in ST and SS (**b**) and between both genotypes at 2, 6, 12 and 24 h (**c**), respectively

A summary of RNA-Seq data is shown in Additional file 1: Table S1, and revealed that the RNA-Seq datasets are robust quality and reliable results were obtained from the transcriptome assembly. A high correlation between biological replicates was observed (R^2^ > 0.92) for all treatments (Additional file 2: Figure S1), which indicated that the biological replicates were reliable in this study. The principal component analyses (PCA) suggested that the PC1, which represents the difference between the control (0 h) and treatment groups (2, 6, 12, 24 h), captured most of the variance in the data (Additional file 3: Figure S2). The samples from the time points could be separated by the PC2, which captured less variance in the data (Additional file 3: Figure S2). In addition, the mRNA expression data also suggested that the expression difference between control and treated groups are dramatically greater than that between different time points of treatment. The FPKM (fragments per kilobase of transcript per million fragments mapped) value of 10 randomly selected genes in the two genotypes at the four time points under salt treatment (80 comparisons, 10 × 2 × 4) was well correlated with its relative expression via qPCR (r^2^ = 0.86; Additional file 4: Figure S3). Figure 2a summarizes the total number of up- and down-regulated genes of both genotypes under salt stress, revealing that the expressions of a large proportion of salt-responsive genes were induced at 12 h in both genotypes after salt treatment, and the total number of salt-responsive genes in SS was much larger than in ST after exposure to salt stress for 2 h.

#### Gene set enrichment analysis for the DEGs in response to salt stress

**Fig. 3:**
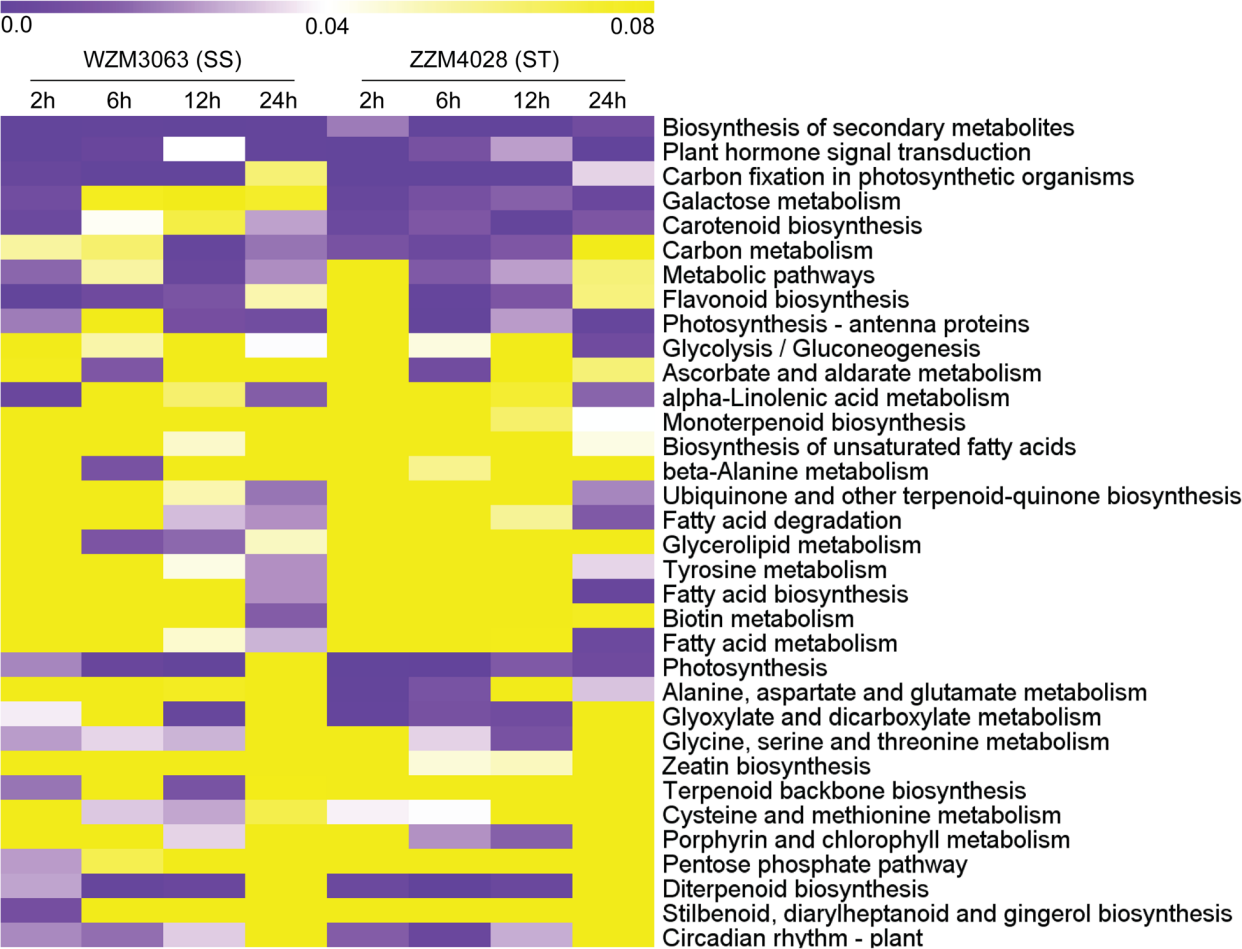
Heatmap of enriched KEGG pathways of the DEGs in ST and SS at different salt stress time points

Compared with control (0 h), a total of 2227/5485, 5881/6928, 7969/7014, and 5745/6660 genes were identified to be differentially regulated in ST/SS at 2, 6, 12, and 24 h, respectively (Fig. 2a). Many salt-responsive genes detected at the different sampling times under salt stress were genotype specific and time specific, which may have contributed to the phenotypic differences in salt tolerance between ST and SS (Fig. 2b and c). Gene enrichment analysis of the DEGs based on the KEGG revealed that these genes were mainly involved in several categories, including “amino acid metabolism”, “carbohydrate metabolism”, “global and overview maps”, “lipid metabolism”, and “metabolism of terpenoids and polyketides” (Additional file 1: Table S2). Under salt stress, biosynthesis of secondary metabolites and plant hormone signal transduction were enriched significantly in both genotypes at all salt stress time points (Fig. 3). In particular, several pathways were preferably enriched with DEGs in ST genotype at different time points, including galactose metabolism, glycolysis/gluconeogenesis, glyoxylate and dicarboxylate metabolism, alanine, aspartate and glutamate metabolism, porphyrin and chlorophyll metabolism, carotenoid biosynthesis, etc. (Fig. 3). GO enrichment analysis of the DEGs showed that a large number of DEGs were involved in some important GO terms which are known to be associated with salt tolerance in plants, such as oxidation-reduction process, response to hormone, response to abiotic stimulus, oxidoreductase activity, and protein kinase activity (Additional file 5: Figure S4). Moreover, GO analysis also showed apparent genotype- and time-specific results, revealing that some important biological processes, cellular processes and metabolic activities occurred in differently in the 2 genotypes under salt stress (Additional file 5: Figure S4). For instance, apoplast, cellular polysaccharide metabolic process, glucan metabolic process, monooxygenase activity, regulation of cellular protein metabolic process, regulation of protein metabolic process, and response to abiotic stimulus were highly or uniquely enriched in ST genotype at different sampling times under salt stress (Additional file 5: Figure S4).

#### Constitutively active genes of ST and SS genotypes in responses to salt stress

As shown in Fig. 2b, there were 1275 and 1946 DEGs that were commonly identified in four treatment groups of ST and SS, respectively, while 374 and 1045 DEGs were exclusively found in ST and SS under salt stress, respectively. It is worth noting that 59 genes which were exclusively and highly up-regulated (FPKM > 10 during at least one time point) in ST at all time points after salt treatment were identified as candidate genes for improved salt tolerance in ST (Additional file 1: Table S3). Of these, several genes of regulatory function were notable, such as ABC transporter (LOC105170264), β-glucosidase (LOC105173929), cytochrome P450 (LOC105161642), dehydration-responsive element-binding protein (LOC105157670), UDP-glycosyltransferase (LOC105171082) (Additional file 1: Table S3). Although different sets and numbers of genes were expressed by the two genotypes, a total of 901 genes (206 up- and 695 down-regulated) were constitutively active in both genotypes and represent the core genes associated with response to salt stress despite tolerance levels in sesame, including a lot of heat-shock proteins, heat-shock transcription factors, late embryogenesis abundant proteins (LEAs), probable protein phosphatase 2C (PP2Cs) (Fig. 2b, Additional file 1: Table S4). Especially, among these core genes, a total of 101 transcription factors were identified which grouped into 31 families, including AP2-EREBP, bHLH, bZIP, HB, MYB, NAC, and their diverse expression patterns indicate their important regulatory roles in salt stress responses (Additional file 1: Table S4, Additional file 6: Figure S5). Alterations in their expression could significantly affect translation of other proteins under salt stress. Based on the GO enrichment analysis, the 901 core genes were found to participate in some important biological processes, including response to auxin, response to hormone, cellular glucan metabolic process, response to abiotic stimulus, polysaccharide metabolic process, protein phosphorylation, and glutamine biosynthetic process (Additional file 1: Table S5). Moreover, many genes were enriched in molecular functions related to kinase activity, protein kinase activity, hydrolase activity, hydrolyzing O-glycosyl compounds and hydrolase activity, acting on glycosyl bonds (Additional file 1: Table S5).

#### Metabolic analysis of ST and SS in response to salt stress

**Fig. 4:**
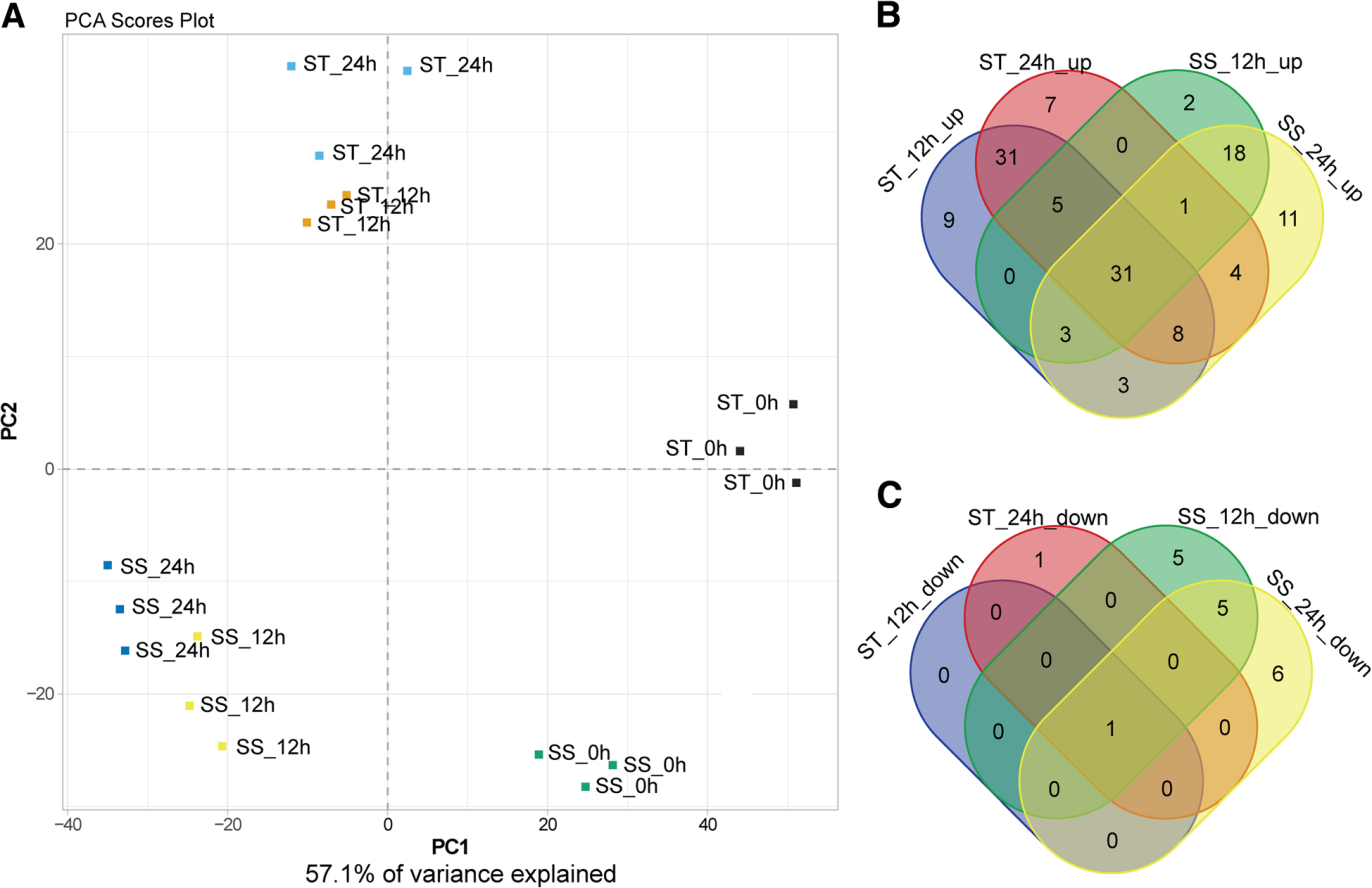
PCA clustering based on metabolome data (**a**) and venn diagram of increased (**b**) and decreased (**c**) metabolites in ST and SS under salt stress

In total, 282 metabolites were detected in sesame by liquid Chromatograph Mass Spectrometer (LC-MS) and Gas Chromatography Mass Spectrometer (GC-MS), including a lot of primary and secondary metabolites, such as amino acids, lipids, organic acids, sugars, alkaloids, amines, flavonoids, terpenoids etc. Compared with control (0 h), 145 measured metabolites were identified to be significantly changed in ST or SS genotypes at 12 h or 24 h after salt stress. In order to find out if the metabolomics profiles of treated and control plants differ from each other PCA was performed. The PCA shows a clear separation between the time points by PC1 and the separation of genotypes can be observed by PC2 (Fig. 4a). In addition, the biological replicates were projected closely in the space, which indicated a good correlation between replicates. The numbers of metabolites that were significantly increased and decreased in one or both genotypes in response to salt stress exposure are presented in Fig. 4b and c, revealing that most of the measured metabolites are increased after salt treatment. Through metabolic pathway mapping, the changed metabolites in response to salt stress were mainly involved in amino acid metabolism, raffinose family oligosaccharides metabolism, citrate cycle (TCA cycle), glycolysis/gluconeogenesis and urea cycle, suggesting that these metabolic pathways may play important roles in rapid adaptive response to salt stress in sesame (Additional file 7: Figure S6).

In total, 31 increased and only one decreased metabolites were shared by both genotypes and represent the core metabolites in sesame response to salt stress (Fig. 4b and c, Additional file 1: Table S6). Among them, the accumulation levels of 13 free amino acids, 8 sugars (containing sugar derivatives) and abscisic acid (ABA) were significantly increased in both genotypes under salt stress, indicating their important roles in osmotic regulation contributing to salinity tolerance of sesame (Additional file 1: Table S6). As shown in Fig. 4b and c, a lot of changed metabolites at 12 h and 24 h under salt stress were also genotype specific. Particularly, 47 measured metabolites showed exclusively significant increase at 12 h or 24 h in ST genotype under salt treatment, including 7 free amino acids, 6 dipeptides, 10 sugars and sugar derivatives, 11 organic acids, caffeic acid, spermidine, etc. (Additional file 1: Table S7). Notably, the aspartate content increased by 13.0-fold and 12.5-fold in ST at 12 h and 24 h under salt stress, respectively, while the glutamate content increased by 17.9-fold and 26.4-fold in ST at 12 h and 24 h under salt stress, respectively (Additional file 1: Table S7).

### Association of transcriptomic and metabolomic changes involved in crucial biological pathways

To obtain more informational perspective on the physiological changes in response to salt stress in sesame, we focused on the connection between gene expression and salt-responsive metabolites changes. In general, several genes encoding key enzymes were involved in either biosynthesis or degradation process of crucial metabolites, suggesting that these genes could be valuable targets for improved salt tolerance in sesame.

### Amino acid metabolism in sesame during salinity stress

**Fig. 5:**
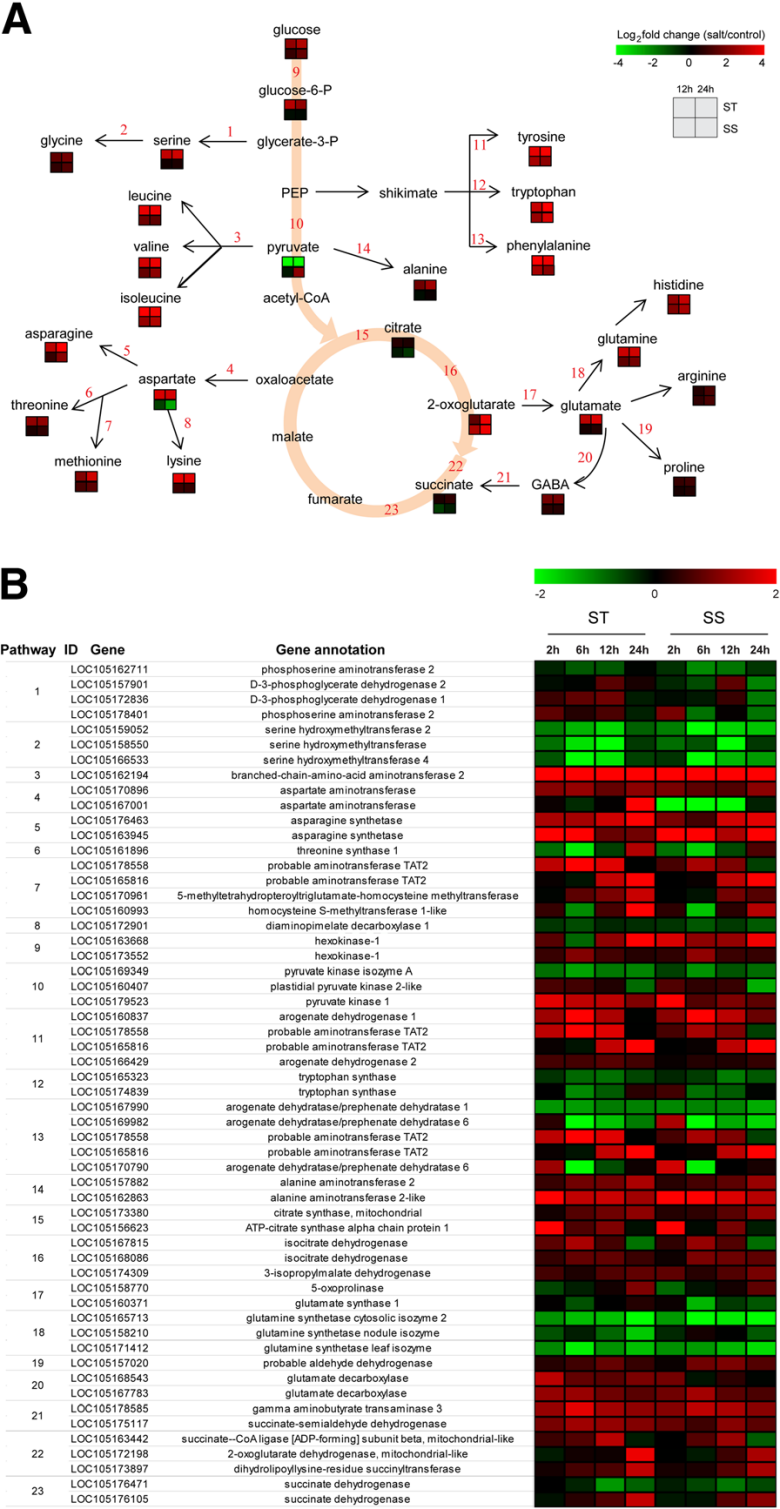
Adaptive changes in amino acid metabolism in ST and SS during salinity stress. **a** Amino acid metabolic pathways and the general pattern of metabolites changes associated with the pathways in ST and SS at 12 h and 24 h after salinity stress. **b** Expression changes of the genes involved in metabolic pathways in ST and SS at different salt stress time points. The pathway ID column indicates the gene product in relation to metabolic pathway

It is noteworthy that pathways involved in amino acid metabolism were enriched significantly in both transcriptomic and metabolomic data. Figure 5 A shows the amino acid metabolic pathways and the general pattern of the relative changes of related metabolites in ST and SS genotypes under salt condition. The content of most free amino acids considered as osmotic regulatory substances, was significantly increased in one or both genotypes, implying that the accumulation of free amino acids is crucial in sesame response to salt stress (Fig. 5a). Obviously, the induction rate of accumulation of many free amino acids (including alanine, asparagine, aspartate, glutamate, glutamine, glycine, isoleucine, leucine, lysine, methionine, ornithine, phenylalanine, proline, serine, threonine, tyrosine, and valine) was more higher in ST than in SS, which may be a positive feature for withstanding salinity stress in ST genotype (Fig. 5a). The expression patterns of the genes involved in the amino acid metabolic pathways were presented in detail in Fig. 5b. In the pathways leading to the strong salt-induced accumulation of arginine, glutamine, glycine, methionine, ornithine, phenylalanine, and tyrosine, most of genes involved in the biosynthesis of these amino acids were up-regulated in both genotypes under salt stress, although there was a trend of down-regulation among some enzymes of these pathways (Fig. 5b). In particular, several genes involved in the biosynthesis of aspartate, threonine, methionine, tyrosine and phenylalanine, showed higher changes in ST than in SS at 12 h or 24 h after exposure to salt, such as aspartate aminotransferase (LOC105167001), threonine synthase 1 (LOC105161896), 5-methyltetrahydropteroyltriglutamate-homocysteine methyltransferase (LOC105170961), homocysteine S-methyltransferase 1-like (LOC105160993), probable aminotransferase TAT2 (LOC105178558). LOC105162194 encoding a branched-chain-amino-acid aminotransferase 2 which is involved in the different pathways leading to the synthesis of isoleucine, leucine and valine, was significantly and continuously up-regulated in both genotypes after salt stress (Fig. 5b). This adjustment of genes may be important to maintain the biosynthesis of some amino acids under stress. Some genes encoding enzymes of serine hydroxymethyltransferase, tryptophan synthase, and glutamine synthetase tended to be down-regulated in both genotypes at almost all time points after salt treatment, so the greatly increased accumulation of serine, tryptophan, and glutamine could be mainly due to greatly reduced degradation (Fig. 5b). Moreover, 4-Aminobutanoic acid (GABA) as an important derivative from glutamate was observed strongly salt-induced in ST genotype, while the expressions of genes for glutamate decarboxylase involved in GABA biosynthesis pathway were up-regulated remarkably in ST under salt stress, especially at 24 h. In general, the integration of both omics data revealed that amino acid metabolism is strongly enhanced for contributing to salt tolerance in sesame, especially in ST genotype.

### Alterations in sucrose and raffinose family oligosaccharides metabolism after exposure to salt

**Fig. 6:**
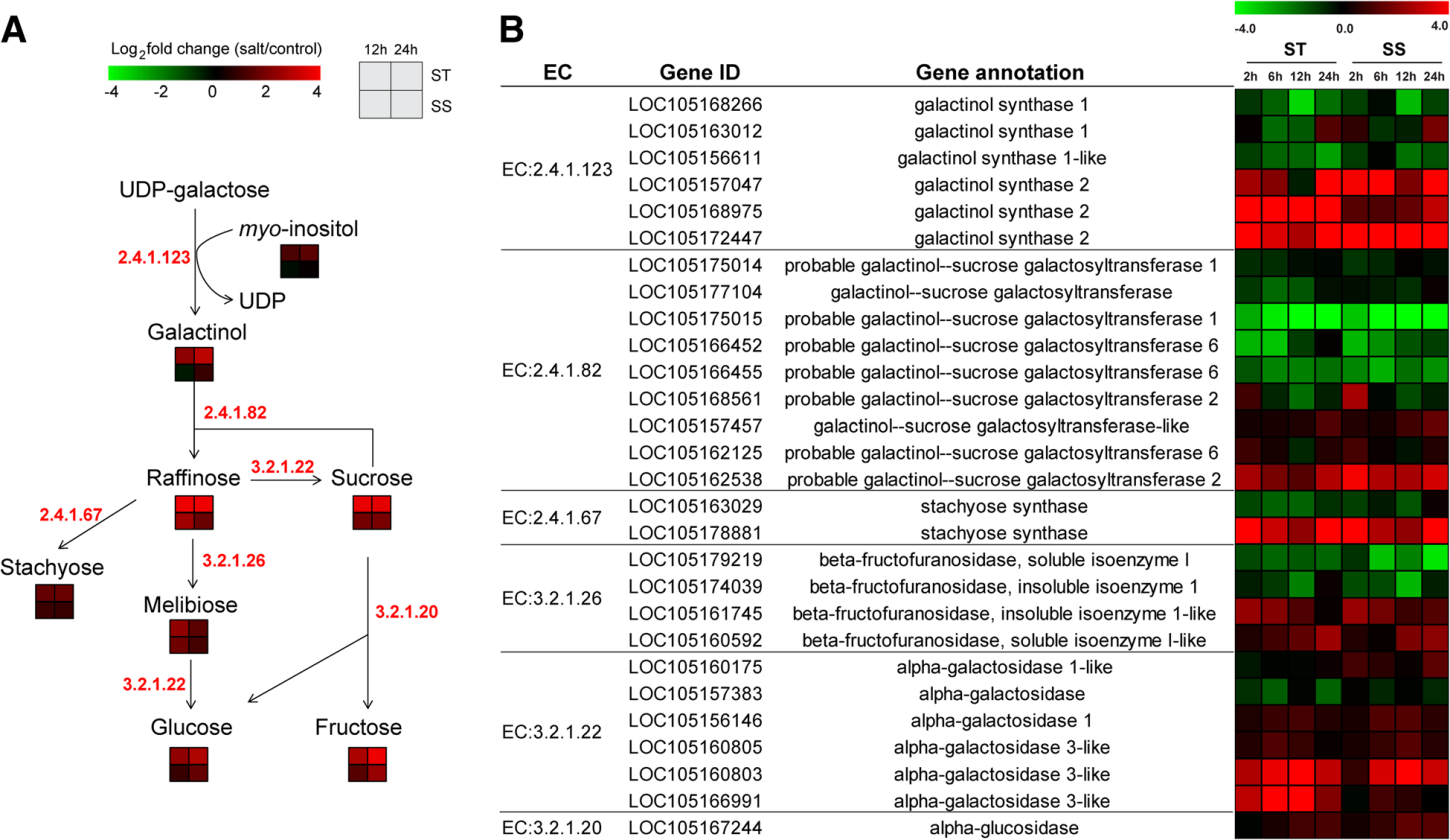
Alterations in RFOs metabolism in ST and SS response to salt stress. **a** RFOs metabolic pathways and the general pattern of metabolites changes associated with the pathways in ST and SS at 12 h and 24 h after salt stress. **b** Expression changes of the genes involved in metabolic pathways in ST and SS response to salt stress

The raffinose family oligosaccharides (RFOs), such as raffinose and stachyose, play an important role in protection against abiotic stress [25]. Figure 6a showed the schematic presentation of sucrose and RFOs biosynthetic pathways in sesame. Interestingly, most of metabolites accumulation involved in sucrose and RFOs metabolism including galactinol, myo-inositol, raffinose, stachyose, sucrose, melibiose, fructose, glucose, were significantly increased in the two genotypes under salt stress, but their fold changes were consistently higher in ST than in SS (Fig. 6a). The expression patterns of most genes involved in the same pathway were similar in the two contrasting genotypes in response to salt stress (Fig. 6b). Three genes encoding galactinol synthase 2 (EC 2.4.1.123) that plays a key regulatory role in the synthesis of galactinol were up-regulated in both genotypes under salt stress, but the expression of gene (LOC105168975) was increased more higher in ST compared with SS under salt treatment (Fig. 6b). Galactinol-sucrose galactosyltransferase (EC 2.4.1.82) and stachyose synthase (EC 2.4.1.67) catalyze the key steps for the synthesis of raffinose and stachyose. The accumulation of raffinose and stachyose which were strong salt-induced both in ST and SS genotypes could be primarily attributed to strong salt-induced expression of a galactinol-sucrose galactosyltransferase gene (LOC105162538) and a stachyose synthase gene (LOC105178881) by salt stress. In addition, most of genes involved in the pathways leading to the accumulation of sucrose, melibiose, fructose and glucose were also up-regulated in both genotypes in response to salt stress. Therefore, these results suggested that the salt tolerance ST genotype could be related to the stronger ability to regulate carbohydrates accumulation in sucrose and RFOs metabolism.

## Discussion

Salt stress as one of the major environmental factors that influence the worldwide distribution of plants, limits growth and productivity of plants, and threatens food security. It is necessary to identify major salt response and tolerance mechanisms in plant to improve crop salt stress resistance. Here, we tracked the transcriptomic and metabolomic changes in sesame seedlings of two contrasting genotypes under salt stress over a period of 24 h, which could provide us with a unique opportunity to gain insights into the candidate genes and metabolites involved in metabolic pathways underlying salt tolerance in sesame.

### Osmotic adjustment under salt stress

High salt level has osmotic effect on plant cells [5, 7]. When plants are subjected to salt stress, the osmotic stress immediately reduces cell expansion in root tips and young leaves, and drastically reduces stomatal conductance [7, 26]. Many plants can accumulate compatible solutes to maintain a stable osmotic pressure and protect membranes and proteins from degradation by mediating osmotic adjustment [27, 28]. Consistent with previous findings, our research showed that strong salt-induced accumulation was observed for most of amino acids, sugars (maltose, melibiose, raffinose, stachyose, sucrose, tagatose and trehalose), and sugar alcohols (erythritol, inositol, xylitol) in sesame, especially in ST genotype (Figs. 5a and 6a, Additional file 1: Tables S6 and S7), while many of them have been recognized as osmolytes in the cytoplasm under salt stress in plants [29, 30]. Recent studies of genes controlling the synthesis or metabolism of several osmolytes have suggested their important roles in salt tolerance, such as *mtlD* [13]*, P5CS* [15], *OTS* [31]. In addition to function as compatible solutes, amino acids could serve as the precursors of secondary metabolites protecting plants from stresses [32, 33]. Previous reports showed that some metabolites involved in plant defense responses were synthesized from amino acid metabolic pathways, which demonstrated that amino acid metabolism play key functions in response to stress in plants [34, 35]. Specifically, the glutamate content in ST increased over 17.9-fold under salt stress, implying that glutamate may serve as a key player in salt tolerance in sesame. Glutamate is the precursor of proline and GABA, and one of the tripeptides in glutathione, as well as serves as substrate for converting to α-ketoglutaric acid which is an important intermediate of carbon and energy metabolism [36]. Here, we observed the induction of several genes involved in amino acid metabolism, sucrose and RFOs metabolism, as well as other sugars biosynthesis, which was consistent with our observation of salt-induced accumulation for special metabolites (Figs. 5b and 6b). In short, our transcriptomic and metabolomic analysis strongly revealed that salt-induced accumulation of these important metabolites through synthesis or metabolism pathways may contribute directly to improving salt tolerance of sesame.

### Reactive oxygen species (ROS) homeostasis during salinity stress

In plant, salt stress causes the accumulation of ROS (such as ^1^O_2_, H_2_O_2_, O_2_·- and HO·,) that result in oxidative damage and programmed cell death [37]. On the other hand, ROS might act as signal molecules to regulate stress responses rapidly [38]. Previous studies reported that the Na + −induced stabilization of SOS1 mRNA is mediated by reactive oxygen species [39]. The connection between SOS pathway and ROS signaling was further demonstrated through the interaction of SOS2 with both NDPK2 (nucleoside diphosphate kinase 2) and catalases [40]. To maintain the ROS homeostasis under stressful conditions, plants have evolved antioxidative defense mechanisms, including ROS scavenging system [41]. During stress, ROS scavenging enzymes such as ascorbate peroxidase, catalase, peroxidase, glutathione S-transferase, and superoxide dismutase, are essential for ROS detoxification [42]. Previous studies have shown that ROS scavenging mechanisms play important roles in protecting plants against salt stress [42–44]. In this study, transcriptomic data revealed that a large number of DEGs both in ST and SS were related to oxidation-reduction process and oxidoreductase activity, suggesting that oxidative stress might be activated at the preliminary stage in response to salt stress. Furthermore, certain salt responsive genes encoding enzymes which involved in ROS scavenging system, including peroxidase and glutathione S-transferase, were strongly up-regulated both in ST and SS under salt stress, implying that regardless of the salt tolerance level, ROS scavenging system is an important protection or tolerance mechanism in sesame adaptive response to salt stress.

### Ionic adjustment in response to salt stress

In plant responses to salt stress, ion homeostasis such as Na^+^ and K^+^ is important for maintaining cytosolic enzymes activities, membrane potential and an appropriate osmotic pressure of cells [45]. The detrimental effect of salt stress on most plant growth and development was mainly caused by Na^+^ toxicity, and so most intensive studies was concentrated on Na^+^ stress signaling, Na^+^ exclusion, and Na^+^/K^+^ transport [46, 47]. The Salt-Overly-Sensitive (SOS) pathway is well known for salt stress signaling and Na^+^ tolerance so far in plant species, such as *Arabidopsis* [46], poplar [48], tomato [49] and wheat [50]. In this pathway, SOS3 (EF-hand calcium-binding protein) could sense the cytosolic calcium signal elicited by salt stress, and interact with and activate a serine/threonine protein kinase SOS2 [51]. The activated SOS2 phosphorylates and activates SOS1 (a Na^+^/H^+^ antiporter at the plasma membrane), which may function to extrude Na^+^ from the xylem parenchyma cells into the apoplastic space of mesophyll cells [47, 51]. In both sesame genotypes, we observed the salt-induction of many genes for the SOS pathway components, including calmodulin-binding proteins, CBL-interacting protein kinases (CIPKs), together with the Na^+^/H^+^ exchangers (NHXs), suggesting that SOS pathway is pivotal regulator of Na^+^ homeostasis in salt tolerance of sesame materials with different salinity tolerance levels. Vacuolar and plasma membrane NHXs are key regulation factors that reduce accumulation of Na^+^ by transporting them across the tonoplast into vacuoles [46, 52]. Transcriptomic data revealed that a gene for sodium/hydrogen exchanger 4, was found to be induced strongly in ST at 2 h after salt stress, suggests that Na^+^ stress signaling could be strongly and rapidly activated in ST when subjected to salt stress. On the other hand, recent studies showed that maintaining a high cytosolic K^+^ to Na^+^ ratio is important for plant cell metabolism and extra K^+^ could mitigate Na^+^ toxicity [45, 53, 54]. Therefore, K^+^ homeostasis is also important for plant withstanding under salinity stress. In this study, salt stress increases the transcript level of some genes encoding K^+^ transporter and K^+^ channel in sesame. Particularly, several genes for K^+^ channel AKT2/3 and KAT3 and K^+^ transporter 6 were induced strongly only in ST genotype under salt stress, indicating that ST genotype may have a stronger ability of K^+^ transport to maintain ion homeostasis in response to salt stress.

### ABA signal transduction

In this study, functional analysis of DEGs by KEGG and GO enrichment analysis revealed that a large number of genes were involved in plant hormone signal transduction and response to hormone under salt stress, highlighting the pivotal roles plant hormones in regulating sesame responses to salinity stress. In particular, the ABA signaling pathway is well known to be associated with responses to salt stress in plants and ABA content increase could help plants to adapt and survive under salt condition by reducing the accumulation of Na^+^ and improving osmotic adjustment [47, 55]. Our metabolomic data showed that the content of ABA was increased dramatically in the two contrasting genotypes after salt treatment in different ways (Additional file 1: Table S6). The genes for 9-cis-epoxycarotenoid dioxygenases, rate-limiting enzymes in the initial steps of ABA biosynthetic pathway, were also significantly up-regulated in both genotypes during salt treatment (Additional file 1: Table S4). In addition, ABA could be released from the ABA glucosyl ester form stored in vacuoles and apoplasts byβ-glucosidase [56]. Here, a gene forβ-glucosidase was found to be exclusively and highly up-regulated in ST genotype under salt stress (Additional file 1: Table S3). Therefore, rapid accumulation of ABA induced by salt stress in SS could be mainly due to enhancing of ABA biosynthesis, but in ST could be partially due to strongly increased ABA biosynthesis and partially released from the ABA glucosyl ester. Recently, the core ABA signaling pathway has been elucidated following with the identification of ABA receptors (PYR/PYL/RCAR protein family), PP2Cs, and subfamily 2 of the SNF1-related kinases (SnRK2s) [57, 58]. SNF1-related kinases could activate ABA-responsive element binding transcription factors (AREB/ABFs) which containing a bZIP-type DNA-binding domain and positively regulating the expression of ABA- or stress-regulated genes, such as *LEAs* [59, 60]. Moreover, it has been confirmed that a bZIP protein could interact with both transcription factor VP1 and ABA-responsive elements and mediate ABA signals [61]. Transgenic plants overexpressing *bZip* or *LEA* alone showed significantly improved tolerance to salt and drought stresses, while co-transfer of *bZip* and *LEA* exhibited an apparent additive effect on stress-tolerance [17, 62]. In this study, we found that a group of *PYLs*, *PP2Cs*, *SnRK2s*, *AREB/ABFs*, *VP1*, and *LEA* genes in sesame were strongly and rapidly induced or inhibited under salt stress, suggesting that these genes may play crucial roles in ABA signal transduction and protecting sesame from damage caused by salt stress (Additional file 1: Table S4). Further studies should focus on the functional analysis of these candidate genes, which is necessary to identify the protein-protein interactions in adaptive response to salt stress and study the genetic basis of natural variation in salinity tolerance of sesame.

### Other salt stress response related metabolites

It is also worth noting that some important metabolites such as GABA, spermidine and caffeic acid, were highly increased in ST when treated with salt stress (Fig. 5a, Additional file 1: Table S7). Recent studies show that GABA, spermidine and caffeic acid play important roles in improving plant tolerance to salt and other stresses [63–65]. GABA is a derivative from glutamate and as a signal molecule, participates in regulating the expression of genes involved in reactive oxygen species (ROS) scavenging in plants under salt stress [63, 66]. The genes encoding glutamate decarboxylase that contribute to GABA biosynthesis were also observed strongly salt-induced in ST genotype, suggesting their important regulatory roles in sesame response to salt stress (Fig. 5b). Spermidine is one of the major polyamines that participate in the regulation of cellular proliferation and differentiation [64]. High elevation of spermidine level is correlated with salt tolerance in plants [67, 68]. Exogenous application of spermidine could improve salt tolerance in plants by maintaining K^+^/Na^+^ homeostasis, enhancing activity of antioxidant enzymes, increasing the osmolytes level and preventing cellular membrane damage [68, 69]. In this study, most of genes involved in spermidine biosynthesis and degradation pathways were inhibited by salt stress, implying that the increased accumulation of spermidine could be mainly attributed to greatly reduced degradation. For caffeic acid, recent studies show that 4-coumarate-CoA ligase can protect plant against adversity stress through enhancing activity of antioxidant enzymes and regulating soluble sugar and proline contents [70, 71]. We also observed that the caffeoylshikimate esterase and 4-coumarate:CoA ligase genes which participate in caffeic acid biosynthesis and degradation, were up-regulated and down-regulated in ST under salt stress, respectively, probably resulting in the accumulation of caffeic acid in sesame as an adaptive response.

## Conclusions

In summary, using our transcriptomic and metabolomic dataset, we successfully identified a lot of candidate genes and metabolites involved in crucial biological pathways underlying salt tolerance in sesame. Our data suggested that the main salt tolerant characters in sesame could be attributed to not only a single gene or metabolite, but a complex regulation and signaling mechanisms, and further studies should focus on how do these candidate genes or metabolites participate in the salt tolerance of sesame.

## Methods

### Plant materials and experimental design

Two sesame accessions of salt-tolerant WZM3063 (ST) and salt-sensitive ZZM4028 (SS) were obtained by salt tolerance evaluation for 490 sesame core collections preserved at the China National Genebank, Oil Crops Research Institute, Chinese Academy of Agricultural Sciences [22]. The sesame seeds were sterilized with 3% sodium hypochlorite for 10 min and washed four times using sterile water. The seeds were germinated on two filter papers with sterile water in an illuminated incubator using a 16/8 h light/dark cycle at 28 °C. Three days later, the uniform seedlings were placed in a box containing half-strength Hoagland solution. 14-day-old seedlings were treated with 150 mM NaCl for different time points. The shoots of treated seedlings were harvested at 0 (control), 2, 6, 12 and 24 h for transcriptome sequencing and the same samples of 0 (control), 12 and 24 h were prepared for metabolite profiling (three biological replicates per treatment). All samples were immediately placed in liquid nitrogen and stored at − 80 °C until use.

### Phenotyping characterization

To assess the salt tolerance of the two genotypes at the germination stage, the sterilized seeds of the two cultivars were germinated on two filter papers with purified water (control) and 150 mM NaCl (with 3 replicates) in a dark illuminated incubator at 28 °C, respectively. Subsequently, the germination rate was recorded at 6 days (d). For salt tolerance assessment of the two cultivars at the seedling stage, the seeds of ST and SS were sown in differently boxes containing nutrient soil for 6 d. Then, the uniform seedlings were subjected to no-stress control and salinity (100 mM NaCl) conditions with three replications for 21 d. Plant survival rate, shoot length, shoot fresh weight, and shoot dry weight of the seedlings under control and salinity conditions were measured. Electrolyte leakage was determined using the method as previous described [72].

### Total RNA isolation and transcriptome analysis

Total RNA of 30 samples was extracted using an EASYspin Plus kit (Aidlab, Beijing, China). RNA-Seq libraries were prepared and sequenced on an Illumina Hiseq X ten platform (Novogene Company, Beijing, China) according to the methods described earlier [73]. After filtering adapters and low-quality sequences, the clean reads were mapped to the sesame genome v.1.0 (https://www.ncbi.nlm.nih.gov/genome/?term=sesamum) using HISAT [74]. Novel transcript prediction and gene expression analysis were carried out as described by Dossa et al [75]. Gene expression level for each sample expressed was according to the FPKM. PCA for evaluating relationships among samples was performed using the software R version 3.1.1. A significant false discovery rate-adjusted *P* value (FDR) < 0.05 based on three biological replicates was used as the empirical parameter to identify the differentially expressed genes (DEGs) [76]. Gene Ontology (GO) and Kyoto Encyclopedia of Genes and Genomes (KEGG) enrichment analysis for the DEGs were carried out by GOseq [77] and KOBAS (2.0) software, respectively. Hierarchical clustering analysis of the DEGs was performed using the MeV 4.9 software [78].

### LC-MS and GC-MS metabolite measurements and data analysis

Metabolites extraction was performed according to metaSysX standard procedures, a modified protocol from Giavalisco et al. [79]. The samples were measured with a Waters ACQUITY Reversed Phase Ultra Performance Liquid Chromatography (LC) coupled to a Thermo-Fisher Exactive mass spectrometer (MS) which consists of an ElectroSpray Ionization source and an Orbitrap mass analyzer as well as with an Agilent Technologies Gas Chromatography (GC) coupled to a Leco Pegasus HT MS which consists of an Electron Impact ionization source and a Time of Flight mass analyzer (metaSysX GmbH, Germany). LC-MS measurements of the aqueous phase permitted the analyses of the secondary metabolites and GC-MS measurements allowed the analyses of the primary metabolites. After alignment and filtration of LC-MS data, annotation was accomplished by matching the extracted data from the chromatograms with library of reference compounds in terms of accurate mass and retention time. For GC-MS data processing and annotation, the Bioconductor package TargetSearch was used to transform retention time to retention index, to align the chromatograms, to extract the peaks, and to annotate them by comparing the spectra and the retention index to the Fiehn Library and to a user created library. The filtered data from all platforms was normalized first to the weight of samples used for extraction and according to sample median intensity group-wise and further combined to a final data matrix. PCA on normalized data was performed using the software R version 3.1.1. Differential metabolites were chosen according to statistically significant (*P* value < 0.05) based on *t* tests using Metaboanalyst 4.0 (http://www.metaboanalyst.ca/). Metabolic Pathways were constructed based on metaSysX’s reference compound database by using VANTED [80].

### qRT-PCR

The same RNA samples for the transcriptome analysis were reverse transcribed using a HiScript II 1st Strand cDNA Synthesis kit (Vazyme Biotech, Nanjing, China) with oligo (dT23) primer. qRT-PCR programs were performed on a LightCycler480 Real-Time PCR System using ChamQTM SYBR® qPCR Master Mix (Vazyme Biotech, Nanjing, China) according to the manufacturer’s instructions. The sesame *Histone H3.3 gene* (*SIN_1004293*) was used as an internal control. The relative expression levels of 10 selected genes were detected with three biological replications and three technical replications using the 2^−∆∆Ct^ method [81]. The gene-specific primers are listed in Additional file 1: Table S8.

## Additional files

Additional file 1: **Table S1.** Summary of RNA-Seq data. **Table S2.** Significantly enriched KEGG pathways of the DEGs in ST and SS in response to salt stress (*P-Value* < 0.05). **Table S3.** List of genes that were exclusively up-regulated in ST genotype at all salt stress time points. **Table S4.** List of the core gene set involved in sesame response to salt stress. **Table S5.** GO terms enriched amongst the core salt-responsive genes in sesame (top 30 terms only). **Table S6.** The core metabolites involved in sesame response to salt stress. **Table S7.** The metabolites increased significantly and exclusively in ST genotype in response to salt stress. **Table S8.** Primers used in qRT-PCR analysis. (XLSX 90 kb)

Additional file 2: **Figure S1.** Pearson correlation analysis between samples. (PNG 790 kb)

Additional file 3: **Figure S2.** PCA clustering based on RNA-Seq data. (PNG 33 kb)

Additional file 4: **Figure S3.** Correlation analysis between qRT-PCR and RNA-Seq data based on log_2_fold change of 10 selected genes. (TIF 38 kb)

Additional file 5: **Figure S4.** GO enrichment of DEGs in ST and SS at different salt stress time points. (TIF 3120 kb)

Additional file 6: **Figure S5.** Expression patterns of the 101 active transcription factors in ST and SS under salt stress. (TIF 4811 kb)

Additional file 7: **Figure S6.** Metabolic pathways of the salt-responsive metabolites in sesame under salt stress. (TIF 3692 kb)

## Abbreviations

ABA: Abscisic acid
AREB/ABFs: ABA-responsive element binding transcription factors
CIPKs: CBL-interacting protein kinases
FPKM: Fragments per kilobase of transcript per million fragments mapped
GABA: 4-Aminobutanoic acid
GC-MS: Gas chromatography mass spectrometer
LC-MS: Liquid chromatography mass spectrometer
LEA: Late embryogenesis abundant protein
NHXs: Na^+^/H^+^ exchangers
PCA: Principal component analyses
PP2C: Probable protein phosphatase 2C
RFOs: Raffinose family oligosaccharides
ROS: Reactive oxygen species
SnRK2s: Subfamily 2 of the SNF1-related kinases
SOS: Salt overly sensitive
SS: Salt-sensitive
ST: Salt-tolerant
