# Supplementary figures and images for: Transcriptome and metabolome analyses of two contrasting sesame genotypes reveal the crucial biological pathways involved in rapid adaptive response to salt stress
Source: BMC Plant Biol. 2019 Feb 11;19:66. doi: 10.1186/s12870-019-1665-6 (PMC6371534; doi:10.1186/s12870-019-1665-6)

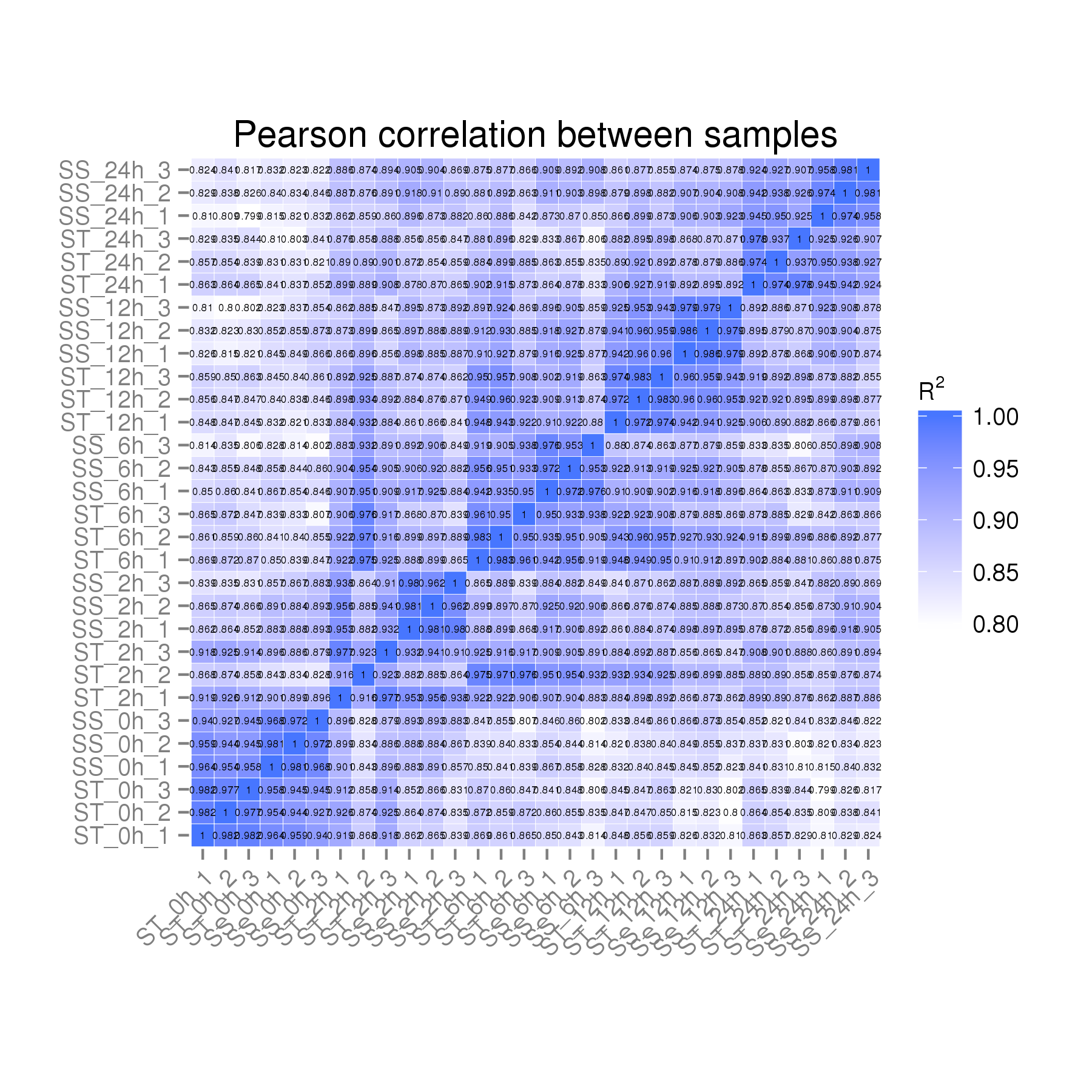

Supplement: Supplementary file 2 — Figure S1. Pearson correlation analysis between samples. (PNG 790 kb) [file 12870_2019_1665_MOESM2_ESM.png]

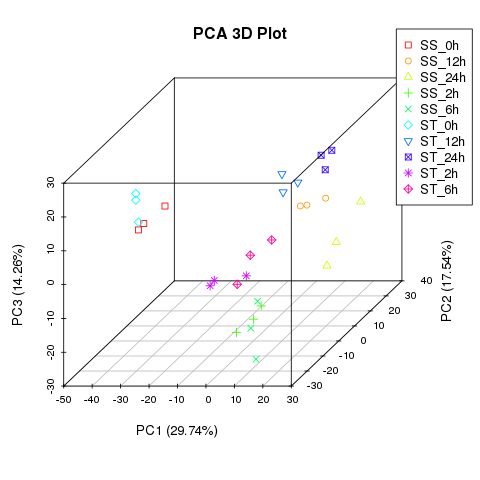

Supplement: Supplementary file 3 — Figure S2. PCA clustering based on RNA-Seq data. (PNG 33 kb) [file 12870_2019_1665_MOESM3_ESM.png]

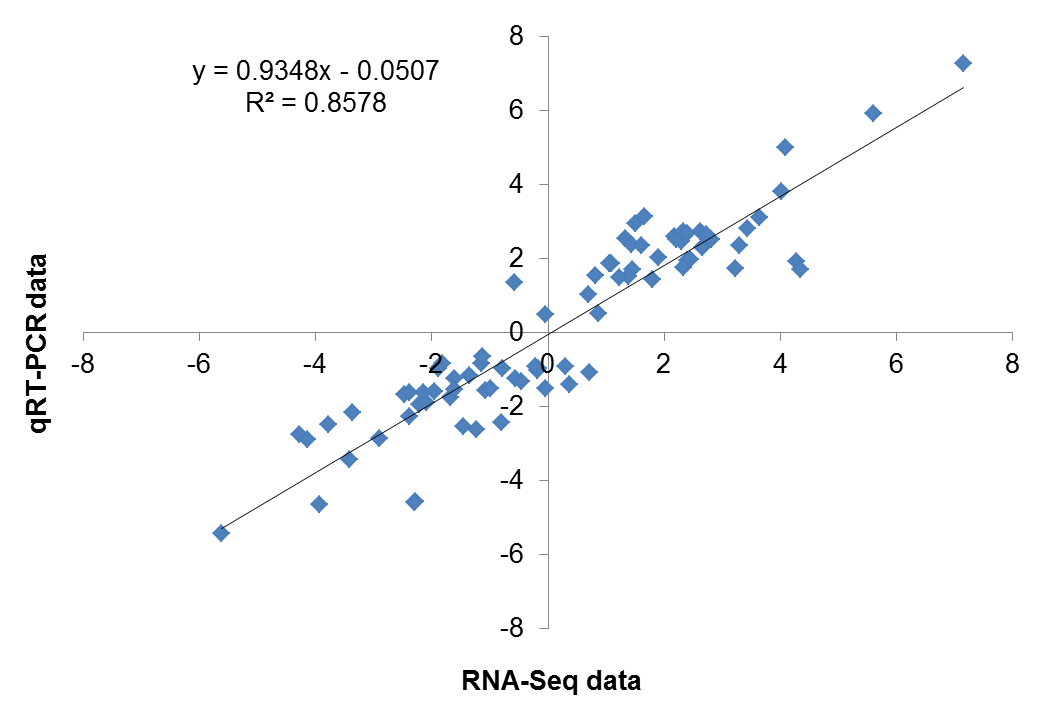

Supplement: Supplementary file 4 — Figure S3. Correlation analysis between qRT-PCR and RNA-Seq data based on log2fold change of 10 selected genes. (TIF 38 kb) [file 12870_2019_1665_MOESM4_ESM.tif]

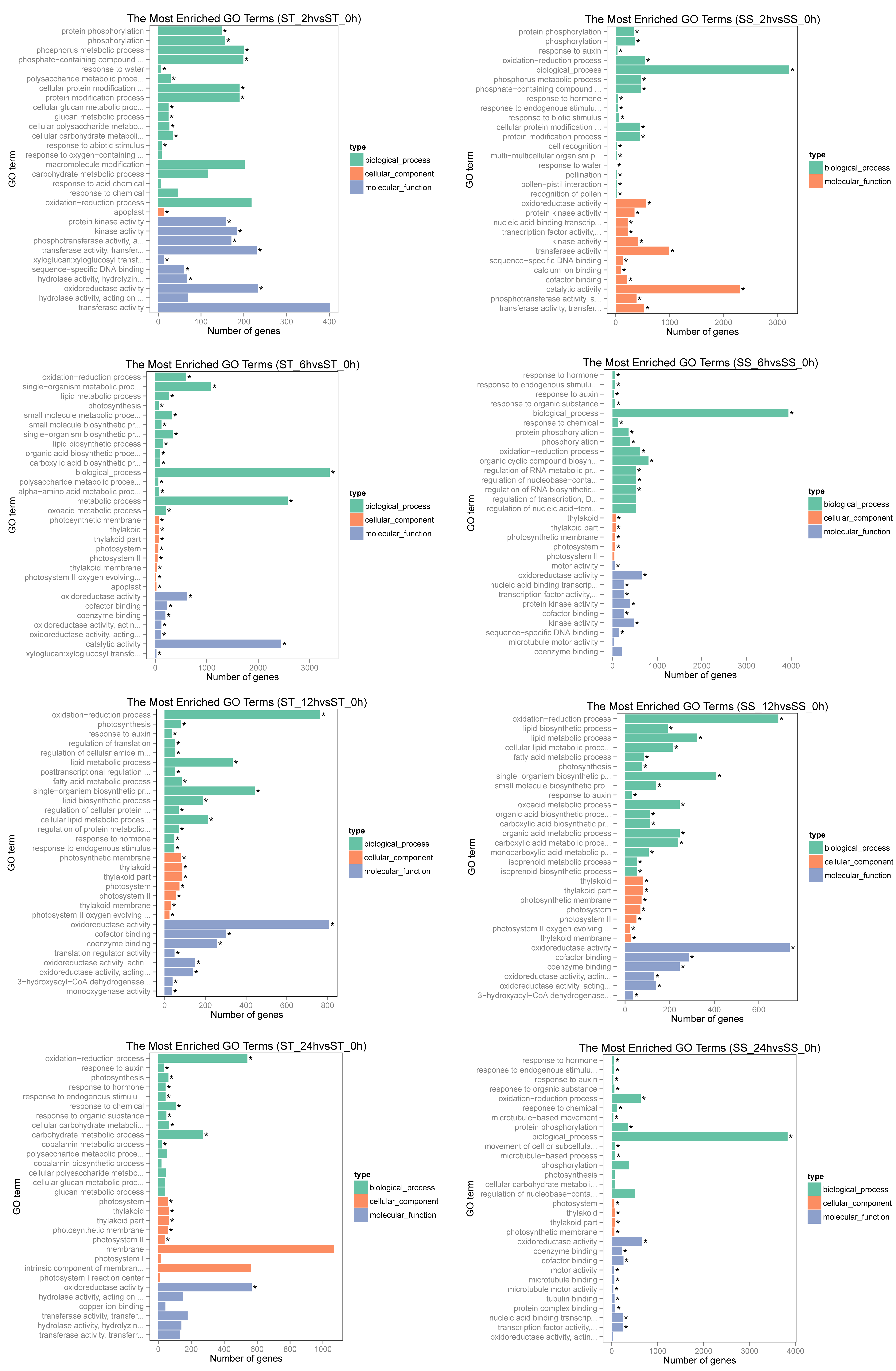

Supplement: Supplementary file 5 — Figure S4. GO enrichment of DEGs in ST and SS at different salt stress time points. (TIF 3120 kb) [file 12870_2019_1665_MOESM5_ESM.tif]

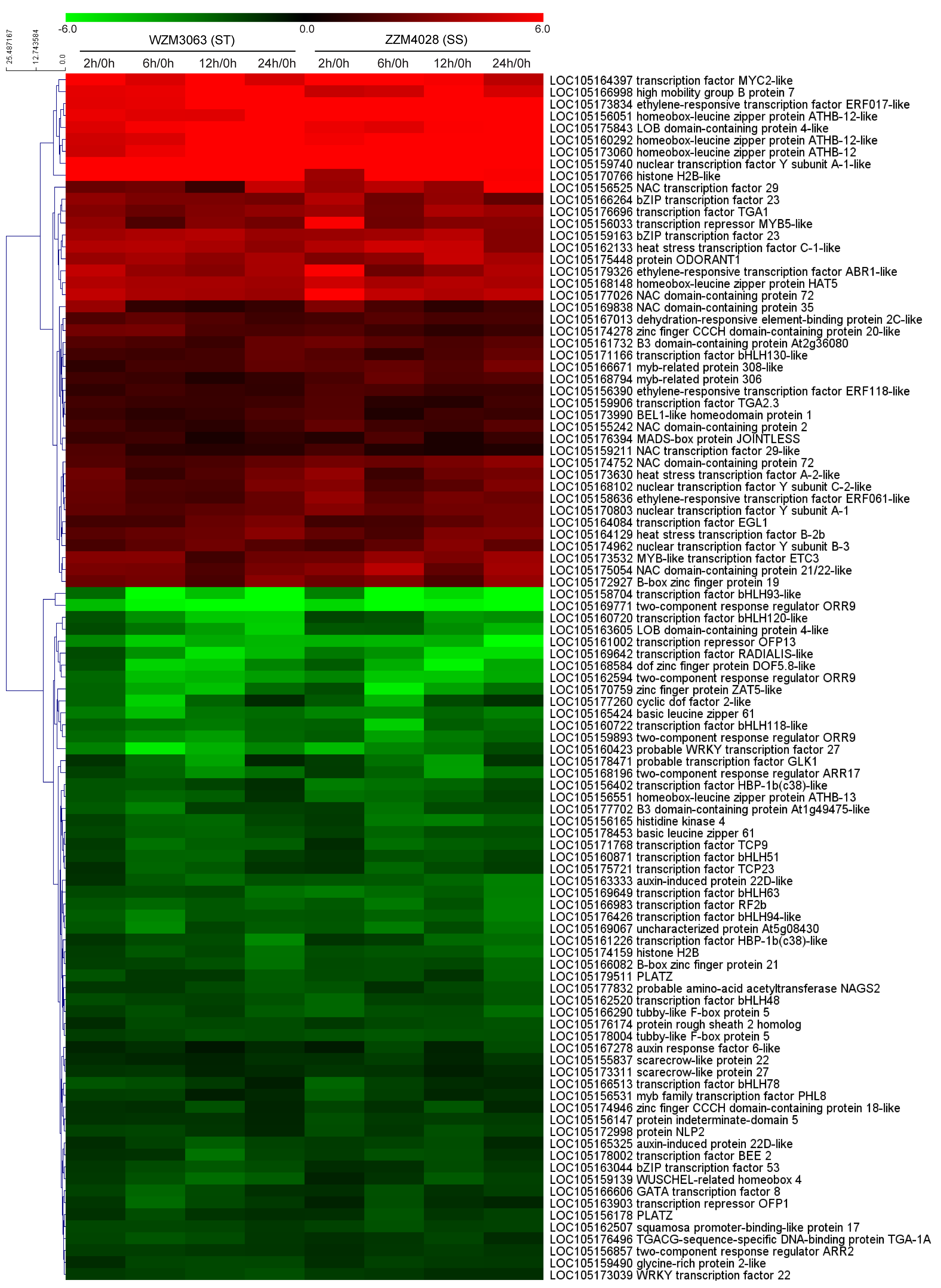

Supplement: Supplementary file 6 — Figure S5. Expression patterns of the 101 active transcription factors in ST and SS under salt stress. (TIF 4811 kb) [file 12870_2019_1665_MOESM6_ESM.tif]

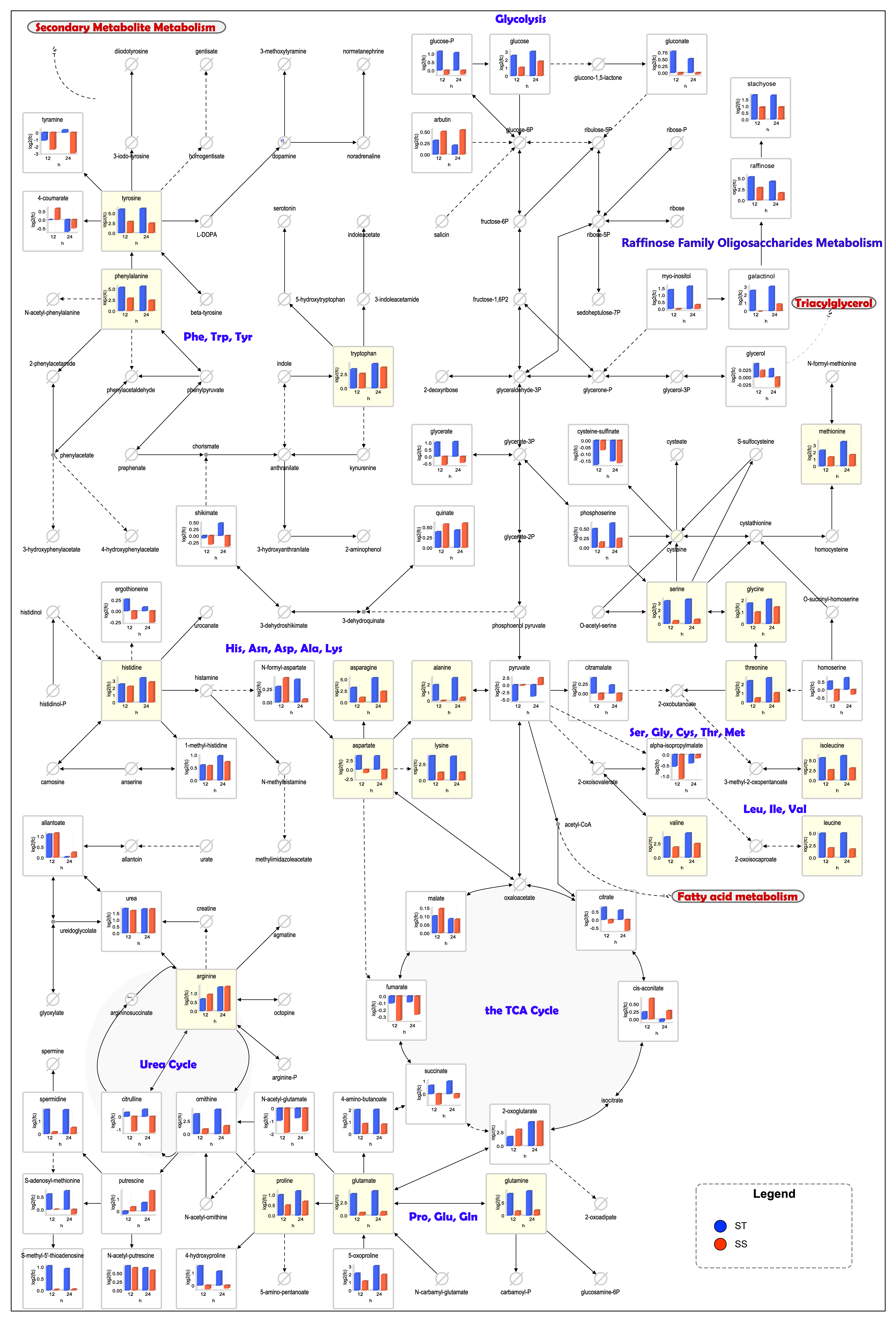

Supplement: Supplementary file 7 — Figure S6. Metabolic pathways of the salt-responsive metabolites in sesame under salt stress. (TIF 3692 kb) [file 12870_2019_1665_MOESM7_ESM.tif]
